# After Exposure to X-Rays, a Transitory Leucocytosis

**Published:** 1913-01

**Authors:** 


After Exposure to X-rays, a Transitory Leucocytosis is frequently observed, differing from the leucocytosis following infection in that the eosinophiles only are increased. Aubertin and Giroux, in *Presse Medicale,* July 13, 1912, report a case in which the eosinophiles reached 65 per cent, the other cells remaining normal in number. Frequent or prolonged exposure causes a destruction of eosinophiles.

